# Outcome measures for economic evaluations and cost‐effectiveness analyses of interventions for people with intellectual disabilities: A methodological systematic review

**DOI:** 10.1111/jar.13056

**Published:** 2022-11-30

**Authors:** Valerio Benedetto, Luís Filipe, Catherine Harris, Naheed Tahir, Alison Doherty, Andrew Clegg

**Affiliations:** ^1^ Synthesis, Economic Evaluation and Decision Science (SEEDS) Group, Health Technology Assessment (HTA) Unit, Applied Health Research hub, University of Central Lancashire Preston UK; ^2^ Methodological Innovation, Development, Adaptation and Support (MIDAS) Theme, National Institute for Health and Care Research Applied Research Collaboration North West Coast (NIHR ARC NWC) Liverpool UK; ^3^ Department of Health Research, Faculty of Health & Medicine Lancaster University Lancaster UK; ^4^ Public Advisers' Forum, National Institute for Health and Care Research Applied Research Collaboration North West Coast (NIHR ARC NWC) Liverpool UK

**Keywords:** cost‐effectiveness, economic evaluations, intellectual disabilities /disability, outcome measures, QALY

## Abstract

**Background:**

Mainstream economic evaluations methods may not be appropriate to capture the range of effects triggered by interventions for people with intellectual disabilities. In this systematic review, we aimed to identify, assess and synthesise the arguments in the literature on how the effects of interventions for people with intellectual disabilities could be measured in economic evaluations.

**Method:**

We searched for studies providing relevant arguments by running multi‐database, backward, forward citation and grey literature searches. Following title/abstract and full‐text screening, the arguments extracted from the included studies were summarised and qualitatively assessed in a narrative synthesis.

**Results:**

Our final analysis included three studies, with their arguments summarised in different methodological areas.

**Conclusions:**

Based on the evidence, we suggest the use of techniques more attuned to the population with intellectual disabilities, such sensitive preference‐based instruments to collect health states data, and mapping algorithms to obtain utility values.

## 1 INTRODUCTION

The challenges facing health and social care services continue to grow, reflecting a rising demand for care (e.g., ageing populations with multimorbid chronic conditions), increasing costs of health and social care interventions and a desire to provide and receive the most effective care (Goodwin et al., 2010; Rawlins, 1999; Woolf et al., 1999). Given constrained funding, difficult decisions have to be made regarding which health and care services should be provided. Increasingly, policy‐making bodies are providing guidance around service provision based on comparisons of the clinical and cost‐effectiveness of different interventions, underpinned by assessments of their relative benefits, harms and costs (National Institute for Health and Care Excellence, 2022). The benefits from health and social care interventions are realised through gains in the length (i.e., reduced mortality) and the quality (i.e., reduced morbidity) of a person's life in different health states (Drummond et al., 2005). Typically, these are combined in economic evaluations through the concept of the quality‐adjusted life years (QALYs), with comparisons of cost‐effectiveness through the cost per QALY gained (Drummond et al., 2005). Although alternatives have been suggested, the simplicity of the QALY and the opportunity to compare across different areas of health and social care have meant that the QALY has become a key instrument for healthcare decision‐making (Weinstein et al., 2009). Despite its widespread use, concerns have been raised about the suitability of QALYs as a health outcome measure for people with specific conditions or receiving certain types of care. People with intellectual disabilities are a group for whom the use of QALYs may not be appropriate, with the risk for an inequitable provision of care.

People with intellectual disabilities have poorer health and die earlier than the general population (Glover et al., 2017; Heslop et al., 2014; LeDeR Programme, 2018). Having an intellectual disability can affect an individual's quality of life (Gilmore & Cuskelly, 2014) and any intervention aimed at reducing challenges incurred by people with intellectual disabilities should be amenable to being evaluated in the scope of a quality‐of‐life assessment. In the estimation of QALYs, to allow comparability across different health areas, health states are typically measured using generic (i.e., not condition‐specific) preference‐based HRQoL instruments, such as the EuroQol 5‐Dimensions (EQ‐5D; National Institute for Health and Care Excellence, 2022). Even though these instruments have been used in some trials to calculate QALYs in the context of intellectual disabilities (Beeken et al., 2013; Melville et al., 2015), these have never been validated in this population. In addition, the techniques usually adopted to elicit the utility values attached to different health states (such as the standard gamble, time trade‐off and visual analogue scale) have become matter of debate in the literature (Fowler et al., 1995; Kahneman, 2009; Nord, 1997; O'Leary et al., 1995; Pettitt et al., 2016).

Fairness and equity represent further sources of concern (Lipscomb et al., 2009). QALYs are estimated by weighing the health gains and life expectancy of people benefiting from a healthcare intervention, but this naturally favours people with acute conditions, who have a bigger margin for improving their health condition and life years. Conversely, patients with chronic conditions are less likely to experience major changes in their health or life expectancy after an intervention (Lipscomb et al., 2009; Nord et al., 2009; Pettitt et al., 2016).

There are also claims that the dimensions considered by generic preference‐based HRQoL instruments, from the scores of which QALYs are typically calculated, are more oriented towards physical health rather than mental health (van Ijzendoorn & Bakermans‐Kranenburg, 2020). For people with cognitive impairments, while the physical component is still relevant (Åström et al., 2020; Brazier et al., 2014), distress and social disability should be key factors to evaluate quality of life, as they can affect the capacity to engage in normal activities as much as physical disabilities (Åström et al., 2020; Chisholm et al., 1997; Wilkinson et al., 1992).

Some of these problems inevitably affect the use of QALYs in economic evaluations of interventions for people with intellectual disabilities, since an intellectual disability is a chronic condition, which impacts on the mental and social skills of affected individuals. Moreover, the range of interactions of people with intellectual disabilities may lead to a new set of methodological problems, since the typical estimation of QALYs, based on the HRQoL of the affected individual, may fail to fully capture externalities (e.g., caregivers and family effects, and long‐term productivity effects), even though methodological advances are being made in this respect (Lamsal, Finlay, Whitehurst, & Zwicker, 2020; Prosser & Wittenberg, 2019).

Since an intellectual disability emerges at birth or in early childhood, children represent a significant sub‐group of people affected by intellectual disabilities (Mencap, 2022a). Children are likely to be supported by caregivers whose life will also be affected by interventions for people with intellectual disabilities (Meltzer & Smith, 2011). Moreover, children may have more problems answering the questions of HRQoL instruments, thus needing a proxy adult respondent whose priorities may differ from those of the child (Payakachat et al., 2014). In addition, traditional generic preference‐based HRQoL instruments used to describe health states and estimate utilities in adults, like the EQ‐5D, have not been validated in younger populations (Sampaio et al., 2021). Equivalent generic preference‐based HRQoL instruments have been developed for these populations, but concerns exist that they may fail to reflect dimensions relevant to specific conditions (Sampaio et al., 2021). Finally, by being at the age of development, children and adolescents have different and more dynamic preferences, which change over time. For them, social integration is a bigger predictor of quality of life, when compared with adults (van Ijzendoorn & Bakermans‐Kranenburg, 2020).

Understanding whether interventions for people with intellectual disabilities are both effective and cost‐effective calls for reliable and standardised methods for performing economic evaluations. The lack of precise and validated measures to evaluate changes in health and quality of life may indirectly discriminate against people with intellectual disabilities (Jahagirdar et al., 2012; Russell et al., 2018), by limiting the methods necessary to examine the cost‐effectiveness of interventions for this population. This methodological gap may add to the discriminations and health inequalities already suffered by people with intellectual disabilities (Feldman et al., 2014; Russell et al., 2018). Finding the right way to evaluate interventions for this population group will contribute to addressing those potentially compounded health inequalities.

Our systematic review aims to identify, assess, and synthesise the different arguments on how the effects of intellectual disability‐related interventions could be measured in economic evaluations, including whether QALYs, as informed by generic preference‐based HRQoL instruments, could be a valid metric in this field. To our knowledge no review has attempted such a task. Previous reviews investigated only one specific methodological area, like the use of instruments to collect health states data (e.g., EQ‐5D; Riemsma et al., 2001), or did not cover the problems of using QALYs to assess mental healthcare interventions in depth (Romeo & Molosankwe, 2010). Our review aims not only to be more up to date but also more general and conceptual, as we group and synthesise the arguments on the use of the QALY and alternative outcome measures. We also consider arguments on those relevant methodological choices, which contribute to the estimation of outcomes in economic evaluations. These arguments pertain to the instruments chosen to collect health states data (like the EQ‐5D), the techniques adopted to elicit utility values, and the perspective and spillover effects considered. For each of these methodological areas, based on the findings from the evidence, we then formalise a set of suggestions to improve the design of future economic evaluations.

## 2 METHODS

The systematic review followed a predetermined protocol (registered on PROSPERO as CRD42021242952) and standard reporting guidance (Page et al., 2021; Table S1).

### 2.1 Search strategy

Our search strategy was informed by the definition of intellectual disability provided by the American Association on Intellectual and Developmental Disabilities, which focuses on an individual's limitations in both intellectual functioning and adaptive behaviour (American Association on Intellectual and Developmental Disabilities, 2022). We also recognised that different terms are used in different countries, such as learning disabilities in the United Kingdom (Mencap, 2022b) and that the terms used have changed over time. The search strategy included similar terms to intellectual disabilities, such as ‘learning or developmental’ disability, or conditions associated with intellectual disabilities, such as ‘Down's syndrome’. Economic terms including ‘cost effectiveness', ‘quality‐adjusted life year’, ‘value’ and ‘outcome’ were also included.

The searches were run on 21st April 2021 on multiple databases: MEDLINE (Ovid); Embase (Ovid); CINAHL Complete (EBSCO); PsycINFO (EBSCO); Cochrane Database of Systematic Reviews and Cochrane Central Register of Controlled Trials (Cochrane Library); International Health Technology Assessment Database; and the NHS Economic Evaluation Database. Searches were limited to the English language and had no date limit. The full search strategies used are presented in Tables S2–S8.

These multi‐database searches were complemented by grey literature searches on three specialist health economic websites: International Society for Pharmacoeconomics and Outcomes Research (ISPOR); International Health Economics Association (iHEA); and the Office of Health Economics (OHE). Due to limitations affecting the search functionality of the above websites, these grey literature searches were run using the Google search engine. The searches included terms similar to those used in the main search (Table S9) and were run by two of the co‐authors (Valerio Benedetto and Luís Filipe) on 16th and 18th June 2021 (respectively).

In addition to the above searches, we also ran backward and forward citation searches to identify other eligible records. With the former searches we checked the reference lists of those studies included during the screening process following the initial searches. With the latter searches, we identified and screened records which cited those studies. The forward citation searches were run on Web of Science, Scopus and Google Scholar on 20th July 2021.

### 2.2 Study selection

An adapted version of the Population, Intervention, Comparator and Outcome model (PICO) guided the study selection, where:

- P: people with intellectual disabilities;
- I: any intervention delivered for people with intellectual disabilities;
- C: any;
- O: the presence of theoretical and empirical arguments describing advantages and disadvantages associated with the measurement, valuation and use of outcome measures for economic evaluations of interventions for people with intellectual disabilities.

We interpreted the Outcome criterion in an inclusive way. This means that we also looked for studies which covered intellectual disabilities as part of a wider set of conditions, for example studies on neurodevelopmental disorders (NDDs). However, in order not to distort the evidence base, we excluded studies which solely focused on other conditions, albeit being common in people with intellectual disabilities, such as autism spectrum disorders (ASDs) and attention deficit hyperactivity disorder (ADHD). No limits were set on the types of settings included. We included any study design but excluded abstracts.

The records obtained by running the multi‐database searches were de‐duplicated and then screened in EndNote by three co‐authors (Valerio Benedetto, Luís Filipe, Catherine Harris) who followed a pre‐piloted screening tool (Table S10). The screening process consisted of two stages:

1. Records were split in three batches assigned to the three co‐authors. Within each batch, the title and abstract of each record was screened by one co‐author, and a random sample (corresponding to 20% of the batch size) cross‐screened by another co‐author;
2. The full text of selected records was then screened independently by two co‐authors.

### 2.3 Data extraction

Data extraction from the selected studies was performed by the same three co‐authors who also validated each other's extractions. These co‐authors used a pre‐piloted Excel template, which included data items specific to the different study designs, such as:

- High‐level details (aim, design);
- Arguments on:
  ○ instruments to collect health states data;
  ○ techniques used to elicit utility values or weights;
  ○ generic and condition‐specific outcome measures;
  ○ and, frameworks for analysis.

The list was reviewed during and following the data extraction process to adapt it to the types of arguments found. For instance, once all the arguments had been extracted, we noticed that a sub‐set of arguments was specifically associated with considering the economic evaluations' perspective and any spillover effects. As such, a specific sub‐category was created to contain these arguments. At the same time, no relevant arguments on the choice of frameworks for the analysis were traced, resulting in the removal of the associated sub‐category.

Any discrepancy in either the study selection or data extraction was resolved through discussions between the three co‐authors, with oversight by another co‐author (Andrew Clegg).

The protocol and this manuscript were reviewed by a member of the public (Naheed Tahir), with her involvement detailed in Table S11.

### 2.4 Quality assessment

In this methodological systematic review, it is the quality of these arguments extracted which is key, rather than the overall quality of the studies wherein the arguments were presented. Traditional checklists which focus on the quality of the studies' design and methodology may not be appropriate to review theoretical or qualitative evidence (Campbell et al., 2014; Lorenc et al., 2014). Consequently, we did not perform a formal quality assessment of the included studies, but the quality of the arguments extracted was assessed as part of the data synthesis.

### 2.5 Data synthesis

We performed a narrative synthesis to bring together the arguments on different methodological areas: perspective and spillover effects; instruments to collect health states; techniques used to elicit utility values; generic and condition‐specific outcome measures. Informed by this narrative synthesis, we then developed a set of suggestions on each methodological area to help the design of future economic evaluations.

## 3 RESULTS

### 3.1 Search results

In total 9273 records were identified from our database searches, of which 3179 were duplicates. Following title and abstract screening, the full texts of 52 records were then screened, as selected from the initial searches (*n* = 7), backward (*n* = 40) and forward (*n* = 3) citation searching, and grey literature searches (*n* = 1). One record was identified from looking at the searches conducted as part of another ongoing review (Benedetto et al., 2022).

The final analysis included three studies, of which two (Lamsal et al., 2020; Russell et al., 2018) came from the initial searches and one (Lamsal & Zwicker, 2017) from the backward citation searches. The reasons behind the exclusion of the other 49 records are described in Table S12.

**FIGURE 1:**
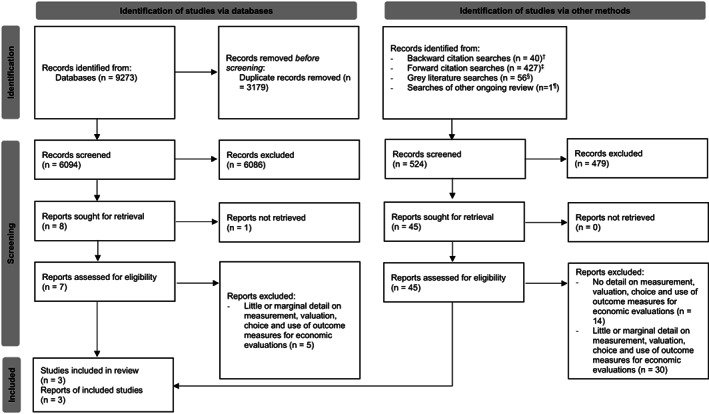
Flowchart reporting search and screening processes identifying included studies. Adapted from: Page et al. (2021). For more information, visit: http://www.prisma‐statement.org/. ^†^Records identified through the backward citation searches were all full‐text screened. Most of these (36 out of 40) were included in the scoping review by Lamsal et al. (2020), which considered 35 types of neurodevelopmental disorders. We screened their full text to avoid any risk of missing potentially relevant studies. ^‡^Records identified through the forward citation searches were screened firstly in terms of their title/abstract and, if relevant, also in terms of their full text. ^§^This figure is approximate as the number of results retrieved by the search engine tends to rapidly vary. ^¶^This record was screened and included in another ongoing systematic review and was deemed relevant for this review too.

The study selection process is summarised in the preferred reporting items for systematic reviews and meta‐analyses (PRISMA) flowchart (Page et al., 2021) presented in Figure 1.

### 3.2 Overall summary of included studies

The included studies were published between 2017 and 2020. One was a methodological study (Lamsal & Zwicker, 2017), another was a scoping review (Lamsal et al., 2020), and the remaining one was a qualitative evaluation (Russell et al., 2018).

One of the studies focused exclusively on people with intellectual disabilities (Russell et al., 2018), while the other two encompassed intellectual disabilities as part of different types of NDDs examined (Lamsal et al., 2020; Lamsal & Zwicker, 2017).

The arguments extracted from the included studies mainly concerned the strengths and limitations of instruments to collect health states data (from all three studies, 100.00%). The study by Lamsal and Zwicker (2017) was the only included study which provided arguments on the use of generic and condition‐specific outcome measures, perspective and spillover effects, and techniques used to elicit utility values (Table 1).

**TABLE 1:** Summary of included studies (*n* = 3)

First author (year)	Aim	Design	Were any of the extracted arguments relevant to …				
			Perspective and spillover effects?	Instruments to collect health states data?	Techniques used to elicit utility values?	Generic outcome measures?	Condition‐specific outcome measures?
Lamsal and Zwicker (2017) (Lamsal & Zwicker, 2017)	To discuss challenges in the context of outcome measurement, costs, caregivers and family effects in economic evaluations of interventions for children with NDDs^a^.	Methodological study.	Yes	Yes	Yes	Yes	Yes
Lamsal (2020) (Lamsal, Finlay, Whitehurst, & Zwicker, 2020)	To discuss the use of generic preference‐based health‐related quality of life instruments in research involving children with NDDs.	Scoping review.	No	Yes	No	No	No
Russell (2018) (Russell et al., 2018)	To produce a qualitative evaluation of the use of the EQ‐5D^b^ administered to people with a mild to moderate intellectual disability and type 2 diabetes.	Qualitative evaluation.	No	Yes	No	No	No
Yes *n* (%)			1 (33.33)	3 (100.00)	1 (33.33)	1 (33.33)	1 (33.33)

*Note*: References: Lamsal et al. (2020); Lamsal and Zwicker (2017); Russell et al. (2018).

^a^ NDD: neurodevelopmental disorder.

^b^ EQ‐5D: EuroQol 5‐Dimensions.

### 3.3 Synthesis of arguments

#### 3.3.1 Perspective and spillover effects

As underlined by Lamsal and Zwicker (2017), multiple international guidelines have recommended the inclusion of spillover effects in economic evaluations of healthcare interventions (Canadian Agency For Drugs And Technologies In Health, 2017; Dutch National Health Care Institute, 2016; National Institute for Health and Care Excellence, 2022; Neumann et al., 2016). For example, in the UK, the National Institute for Health and Care Excellence (NICE) (National Institute for Health and Care Excellence, 2022) recommends that all direct health effects for the patients and, when relevant, for the carers should be taken into account, which is likely to be the case in interventions for people with intellectual disabilities. Lamsal and Zwicker (2017) emphasised that interventions for children with NDDs can impact on outcomes which go beyond the health dimensions of the patients, such as the patients' education and employment prospects, as well as the caregivers' time and productivity. In this sense, according to these authors, the adoption of a societal perspective in economic evaluations would be preferred (Lamsal & Zwicker, 2017).

Specifically, Lamsal and Zwicker (2017) identified two types of spillover effects in interventions targeting the health of children, which represent a large sub‐group of people affected by intellectual disabilities (Mencap, 2022a): those impacting on the health and economic conditions of the caregivers (caregiving effects) and those impacting on the family members (family effects). As cited in this study (Lamsal & Zwicker, 2017), formal methods of incorporating these effects in economic evaluations have been proposed by Basu and Meltzer (2005), through a household utility function accounting for different effects on family welfare, and by Al‐Janabi et al. (2016), through multiplier effects which could capture benefits and disbenefits triggered by the interventions. Specifically for children with intellectual disabilities, Arora et al. (2019) used a discrete choice experiment to estimate the monetary value of informal care which is directly based on the preferences of the caregivers (Arora et al., 2019). However, Lamsal and Zwicker (2017) also pointed to the lack of a consensus over the most appropriate methodology to use to incorporate spillover effects, and the scarcity of evidence in the NDDs area.

#### 3.3.2 Instruments to collect health states data

The study by Russell et al. (2018) provided insights on the use of one of the most common instruments to collect health states data, the EQ‐5D, in a trial involving people with intellectual disabilities. The EQ‐5D is a generic preference‐based instrument which assesses HRQoL through five dimensions (mobility, self‐care, usual activities, pain/discomfort, and anxiety/depression). Russell et al. identified some practical issues associated with administering the EQ‐5D to this population. One issue is linked to the wording of the EQ‐5D questions. To answer them, a certain degree of health‐related knowledge is required, which may not always be present in individuals with intellectual disabilities (Russell et al., 2018). Moreover, the need to consider their own health in the current moment (and not in relation to previous periods) may be challenging (Russell et al., 2018). Other issues pertain to the ED‐5D content. The examples given within the EQ‐5D to illustrate the dimensions (for instance, which activities constitute ‘usual activities’), and the difference in the levels to characterise a dimension (for instance, between ‘extreme’ and ‘moderate’ problems), may not always be clear (Russell et al., 2018). Also, people with intellectual disabilities may tend to show adaptability to their own health‐related problems, which may then be reflected in recording fewer difficulties than (the general population or even caregivers would have) expected in their answers to the EQ‐5D (Russell et al., 2018). Using proxies for assessing objective dimensions or changing the way the EQ‐5D questions are asked may solve some issues, but would divert from the standard way the EQ‐5D ought to be administered (Russell et al., 2018). In conclusion, the authors recommended that the EQ‐5D should not be used as the sole measure assessing HRQoL in this population (Russell et al., 2018). In the absence of evidence on the validation of the EQ‐5D in people with intellectual disabilities, the development of a bespoke version of the EQ‐5D was urged (Russell et al., 2018).

Moreover, administering questionnaires like the EQ‐5D to children affected by cognitive and communication difficulties may be challenging (Lamsal & Zwicker, 2017). In a scoping review of studies on children with different types of NDDs including intellectual disabilities, Lamsal et al. (2020) explored whether existing tools capture dimensions relevant to this child population, and how the impact of the relationships with family and peers on their HRQoL could be measured. The authors explored how generic HRQoL preference‐based instruments had been selected in the literature. They found that this choice is typically grounded on the previous use of the instrument in this population, or on the instrument being tailored for children and adolescents, or again on the instrument having been already validated in the adult population. However, these rationales do not allay the need for specific instruments for children with NDDs that can be administered and capture the dimensions relevant to this younger population (Lamsal et al., 2020).

Proxy reporting may be used when direct data collection from those affected by cognitive difficulties is not feasible (Lamsal et al., 2020; Lamsal & Zwicker, 2017); however, their use is debated in the literature. Lamsal and Zwicker (2017) emphasised the advantages of using proxies which could report visible symptoms, while limitations may emerge when subjective assessments, like assigning utility values, are needed (Lamsal & Zwicker, 2017). As identified by the scoping review of Lamsal et al. (2020) for the evaluations of interventions of children with NDDs, visual aids may be used to enhance the children's (and proxies') understanding of their own health states.

#### 3.3.3 Techniques used to elicit utility values

On the techniques used to elicit utility values, Lamsal and Zwicker (2017) underlined the importance of considering the perception skills of children with NDDs, who may find it problematic to understand time‐related choices between different health states posed by standard gamble and time trade‐off methods. However, a potential solution may be to employ mapping algorithms to convert scores from condition‐specific or generic health instruments into utility values which can then be used to estimate QALYs (Lamsal & Zwicker, 2017).

#### 3.3.4 Generic and condition‐specific outcome measures

As an alternative to developing QALYs using generic preference‐based instruments, Lamsal and Zwicker (2017) discussed the adoption of the capability approach (Lamsal & Zwicker, 2017). Rather than assessing what people do, as measured by the typical QALY approach, the capability approach is based on the assessment of what people are capable of doing, which could be more appropriate in specific mental health populations, such as children with NDDs. However, existing instruments based on the capability approach will need to be refined and tested for specific mental health conditions to be used in economic evaluations (Lamsal & Zwicker, 2017). At the same time, condition‐specific outcome measures used in mental healthcare may capture more relevant dimensions compared with generic outcome measures, but their use in economic evaluations is hindered as cross‐area comparisons would be unfeasible (Lamsal & Zwicker, 2017).

## 4 DISCUSSION

### 4.1 Place in the literature

To our knowledge, this is the first systematic review investigating how the effects of interventions for people with intellectual disabilities could be measured in economic evaluations. Before starting this review, we conducted preliminary searches looking for existing similar reviews. The review by Riemsma et al. (2001) (Riemsma et al., 2001) was included in a health technology assessment report investigating generic health status instruments for people affected by cognitive impairment (originated from intellectual disabilities and acquired brain injury). The authors did not find any preference‐based instruments whose validity had been assessed in people with cognitive impairment and could be used in economic evaluations of interventions for this population. While this previous review investigated instruments to collect health states data, our scope was wider as we also investigated other methodological areas instrumental in the design of economic evaluations.

The review by Romeo and Molosankwe (2010) focused on the economic evidence in intellectual disabilities. The authors noted that the use of QALYs to assess the health gains in people with mental health problems can be difficult but did not delve much more into the application of QALY or its alternatives. Also, the searches for this review covered a relatively short timeframe (from 2006 to 2010). Therefore, a more extensive and up‐to‐date review was needed, and our systematic review fills the temporal and conceptual gaps of these previous reviews.

There exists an extensive literature on methodological challenges in conducting economic evaluations of interventions on conditions which are common in people with intellectual disabilities, such as ASDs or ADHD (Brown et al., 2019; Griffin et al., 2008; Knapp & Buescher, 2014; Payakachat et al., 2012, 2014; Sampaio et al., 2021; Tilford et al., 2012; Tilford et al., 2015). In contrast, for methodological challenges affecting economic evaluations of interventions for people with intellectual disabilities specifically, there is a paucity of studies available, as our review reveals. Despite this, the evidence available seems to indicate that mainstream cost‐effectiveness methods, focused on the HRQoL of the patient only (Lamsal & Zwicker, 2017) and on the use of generic HRQoL instruments (Lamsal et al., 2020; Russell et al., 2018), may fail to reflect the needs and preferences of this population group. As such, the use of these methods may misrepresent the value of the interventions for people with intellectual disabilities, and alternative approaches are necessary. For this purpose, we draw on the evidence available to formalise a set of suggestions, which can guide the design of future economic evaluations of interventions for this population group. In doing so, we also consider approaches from the wider mental healthcare literature which may be adaptable in intellectual disability‐related economic evaluations.

### 4.2 Suggestions for measuring the effects of interventions for people with intellectual disabilities in economic evaluations

#### 4.2.1 Perspective and spillover effects

The overarching message emerging from the arguments extracted is that spillover effects over caregivers and family members should be considered in economic evaluations of interventions for people with intellectual disabilities (Lamsal & Zwicker, 2017). This is in line with international guidelines, which recommend their inclusion where relevant (Canadian Agency For Drugs And Technologies In Health, 2017; Dutch National Health Care Institute, 2016; National Institute for Health and Care Excellence, 2022; Neumann et al., 2016). Naturally, this implies the adoption of a societal perspective in economic evaluations. These spillover effects may have health and socio‐economic impacts, which then reverberate through the society as a whole (e.g., on the levels of productivity and healthcare resource use; Tilford et al., 2015). Nevertheless, how to actually incorporate spillover effects in economic evaluations remains a matter of debate in the literature (Al‐Janabi et al., 2016; Arora et al., 2019; Basu & Meltzer, 2005; Lamsal & Zwicker, 2017; Prosser & Wittenberg, 2019).

#### 4.2.2 Instruments to collect health states data

Generic preference‐based instruments used to collect health states data in economic evaluations are generally not deemed sensitive nor practical enough to be administered to people affected by intellectual disabilities (Lamsal et al., 2020; Lamsal & Zwicker, 2017; Russell et al., 2018). Proxy reporting is often used but comes with challenges too (Lamsal et al., 2020; Lamsal & Zwicker, 2017). Of particular relevance to our review is the study by Russell et al. (2018) which highlighted the problems associated with using the EQ‐5D in a sample of people with a mild to moderate intellectual disabilities, and advocated further research looking to adapt the EQ‐5D to meet the cognitive needs of this population.

The problems with adopting the EQ‐5D extend to the general literature on mental healthcare interventions. For example, according to van Ijzendoorn and Bakermans‐Kranenburg (2020), the dimensions included in the EQ‐5D seem to focus more on physical rather than mental health, which may cause discrimination (Crisp, 1991). Dimensions referring to outward behaviour (e.g., aggressive behaviour) and social relationships appear to be neglected (Chisholm et al., 1997; van Ijzendoorn & Bakermans‐Kranenburg, 2020). Moreover, preventive interventions appear to be undervalued. The utility values attached to the EQ‐5D may assign more weight (and thus QALY gains) to interventions targeting improvements in one dimension, where severe problems exist. Lower weight may be assigned instead to preventive interventions targeting smaller gains over multiple dimensions, where moderate problems exist.

These problems are being addressed by recent efforts aiming to develop and validate mental health preference‐based instruments, such as the Recovering Quality of Life‐Utility Index (ReQoL‐UI; Keetharuth et al., 2021). The ReQoL‐UI could potentially be used in economic evaluations of mental healthcare interventions to generate QALYs, which are sensitive to the effects impacting on mental health dimensions.

#### 4.2.3 Techniques used to elicit utility values

In general, the ways utility values are elicited to evaluate mental healthcare interventions is fraught with difficulties, particularly considering that the general population, from whom utility values are normally elicited, may have different perceptions of, and exposure to, the impacts of mental impairments compared with those actually affected (Brazier, 2008; Chisholm et al., 1997; van Ijzendoorn & Bakermans‐Kranenburg, 2020). As explained by Lamsal and Zwicker (2017), adopting traditional techniques which elicit utility values for use in economic evaluations, like the standard gamble and the time trade‐off, may also be problematic when administered to children with NDDs (including those with intellectual disabilities) who may struggle to understand time‐related choices between different health states.

Solutions to overcome these difficulties may lie in the use of mapping algorithms, which convert the scores from condition‐specific or generic health instruments into utility values usable in economic evaluations (Lamsal & Zwicker, 2017).

#### 4.2.4 Generic and condition‐specific outcome measures

The use of generic outcome measures in economic evaluations has clear benefits in terms of allowing comparisons across different health areas, but at the same time it comes with drawbacks. Chisholm et al. (1997) argued that the use of any composite outcome measure runs the risk of missing important information regarding the patients. This was also corroborated by Brazier's (2008) argument that generic outcome measures do not possess the necessary psychometric properties for all conditions.

As the calculation of QALYs normally emphasises health improvements rather than maintaining health (Chisholm et al., 1997; Donaldson et al., 1988; Hernandez‐Villafuerte et al., 2019) and may neglect important mental health dimensions (due to the use of generic preference‐based instruments like the EQ‐5D; Hernandez‐Villafuerte et al., 2019), the cost‐effectiveness of interventions for people with intellectual disabilities may be underestimated when compared with other healthcare interventions in priority‐setting decisions.

At the same time, condition‐specific instruments, which could capture dimensions relevant to people with intellectual disabilities, do not lend themselves to valuation procedures which are required to estimate utility values and, in turn, QALYs which can be used for cross‐area comparisons (Lamsal & Zwicker, 2017).

In this sense, a middle ground between generic preference‐based and condition‐specific instruments is desirable. The development of a mental health preference‐based instrument was advocated by Brazier (2008) and substantiated by the advent of the ReQoL‐UI which could be used to generate mental health‐sensitive QALYs (Keetharuth et al., 2021). This could be a welcome solution, but the ReQoL‐UI validity requires empirical testing in people with intellectual disabilities before being applied in economic evaluations.

Other potential solutions, like the capability approach (Lamsal & Zwicker, 2017), deserve attention but more empirical work is needed in the intellectual disabilities' area to test their promising features.

#### 4.2.5 Set of suggestions

Informed by the above considerations, we indicate the following set of suggestions for the design of future economic evaluations of interventions for people with intellectual disabilities.

*Suggestion 1 (based on 4.2.1)*: Where possible, spillover effects on caregivers and family members from interventions for people with intellectual disabilities should be incorporated in economic evaluations, which would then be based on a societal perspective.

*Suggestion 2 (based on 4.2.2)*: Promising preference‐based measures specifically tailored to capture the effects of mental healthcare interventions (such as the ReQoL‐UI) are emerging. However, their adoption in economic evaluations in the intellectual disabilities' area will need to be tested in this population. At the same time, the limitations in the use of generic preference‐based instruments (e.g., EQ‐5D) to measure HRQoL in people with intellectual disabilities need to be considered.

*Suggestion 3 (based on 4.2.3)*: To value intellectual disability‐related health states and obtain utility values for the estimation of QALYs, mapping algorithms should be considered.

*Suggestion 4 (based on 4.2.4)*: Building on Suggestion 2, estimating QALYs informed by preference‐based measures relevant to and validated in people with intellectual disabilities should be considered and preferred, where possible, to the alternative of estimating QALYs using the scores from generic preference‐based instruments (like the EQ‐5D).

### 4.3 Strength and limitations

The novelty of our systematic review, the first (to our knowledge) to investigate how the effects of interventions for people with intellectual disabilities could be measured in economic evaluations, is a key strength. In addition to collating, assessing and synthesising the available evidence, we also outlined a set of suggestions, which could help the design of future economic evaluations in this field.

One of the limitations of our review is in the small number of included studies. Despite this, in these studies we found multiple arguments pertaining to different methodological areas which then informed our set of suggestions.

We also recognise that, while in this systematic review we focused on the identification and measurement of outcomes, other methodological choices are crucial in the design of economic evaluations (e.g., identification and measurement of costs, choice of the time horizon and modelling techniques).

### 4.4 Further research

Our systematic review highlighted that further studies, developing and testing preference‐based instruments specific to people with intellectual disabilities from which QALYs can be derived, are needed. In this sense, the development of mental health preference‐based instruments like the ReQoL‐UI (Keetharuth et al., 2021) represents progress, but its adaptability and validity in the intellectual disabilities' population needs to be tested. Importantly, any preference‐based instruments should be co‐developed and co‐produced with, and for, people with intellectual disabilities, their families and caregivers.

While our review extracted and synthesised the available evidence on how to measure effects of interventions for people with intellectual disabilities, more problems and challenges are likely to exist and should come to the fore, as occurred for other common comorbid conditions in this population group, such as ASDs or ADHD (Brown et al., 2019; Griffin et al., 2008; Knapp & Buescher, 2014; Payakachat et al., 2012, 2014; Sampaio et al., 2021; Tilford et al., 2012, 2015).

## 5 CONCLUSIONS

In this systematic review we highlighted how, according to the evidence identified, traditional methods to measure the effects of healthcare interventions are likely not to be suitable in economic evaluations of interventions for people with intellectual disabilities. This is due to the heterogenous effects triggered by such interventions, which are likely to impact on the health and socio‐economic status of caregivers and family members as well as on the individuals with intellectual disabilities (Tilford et al., 2015). Moreover, generic preference‐based instruments, typically used to estimate QALYs, are argued not to be practical to be administered to people with intellectual disabilities (Russell et al., 2018).

On the measurement of health states, in absence of valid alternatives, any use of generic preference‐based instruments like the EQ‐5D to describe the health states of people with intellectual disabilities should consider the limitations of this approach (Russell et al., 2018). On the valuation of health states, we suggest that mapping algorithms need to be considered to obtain utility values which would enter the estimation of QALYs (Lamsal & Zwicker, 2017). New research efforts are encouraged to validate recently developed mental health preference‐based instruments like the ReQoL‐UI (Keetharuth et al., 2021) in people with intellectual disabilities, or to develop and test a new preference‐based instrument specific to this population. Past and future efforts would ultimately allow the estimation of QALYs which are reflective of the effects relevant to people with intellectual disabilities, their families and their caregivers.

## CONFLICT OF INTEREST

The authors declare no conflict of interest.

## Supporting information


**Appendix S1:** Supporting Information

## Data Availability

Data sharing is not applicable to this article as no new data were created or analyzed in this study.
