# Supplementary material for: Outcome measures for economic evaluations and cost‐effectiveness analyses of interventions for people with intellectual disabilities: A methodological systematic review
Source: J Appl Res Intellect Disabil. 2022 Nov 30;36(2):230–40. doi: 10.1111/jar.13056 (PMC10099878; doi:10.1111/jar.13056)
Supplement: Supplementary file 1 — Appendix S1: Supporting Information [file JAR-36-230-s001.docx]

**Supporting Information**

Title:

Outcome measures for economic evaluations and cost-effectiveness analyses of interventions for people with intellectual disabilities: a methodological systematic review

**Contents**

[Table S1 Preferred Reporting Items for Systematic Reviews and Meta-Analyses (PRISMA) 2020 Checklist^†^ 2](#_Toc109750365)

[Table S2 Search strategy used in Medline (Ovid) 6](#_Toc109750366)

[Table S3 Search strategy used in Embase (Ovid) 7](#_Toc109750367)

[Table S4 Search strategy used in CINAHL (EBSCO) 8](#_Toc109750368)

[Table S5 Search strategy used in PsycINFO 9](#_Toc109750369)

[Table S6 Search strategy used in the Cochrane Database of Systematic Reviews and Cochrane Central Register of Controlled Trials (Cochrane Library) 10](#_Toc109750370)

[Table S7 Search strategy used in the International Health Technology Assessment Database 11](#_Toc109750371)

[Table S8 Search strategy used in the NHS Economic Evaluation Database 12](#_Toc109750372)

[Table S9 Websites and search string used in the grey literature searches 13](#_Toc109750373)

[Table S10 Screening tool 14](#_Toc109750374)

[Table S11 Guidance for Reporting Involvement of Patients and Public (GRIPP2) table^†^ 16](#_Toc109750375)

[Table S12 Records excluded following full-text screening (n=49) 18](#_Toc109750376)

[References 22](#_Toc109750377)

## Table S1 Preferred Reporting Items for Systematic Reviews and Meta-Analyses (PRISMA) 2020 Checklist^†^

| **Section and Topic** | **Item #** | **Checklist item** | **Location where item is reported** |
| --- | --- | --- | --- |
| **TITLE** | | |  |
| Title | 1 | Identify the report as a systematic review. | Title |
| **ABSTRACT** | | |  |
| Abstract | 2 | See the PRISMA 2020 for Abstracts checklist. | Abstract |
| **INTRODUCTION** | | |  |
| Rationale | 3 | Describe the rationale for the review in the context of existing knowledge. | Introduction |
| Objectives | 4 | Provide an explicit statement of the objective(s) or question(s) the review addresses. | Introduction |
| **METHODS** | | |  |
| Eligibility criteria | 5 | Specify the inclusion and exclusion criteria for the review and how studies were grouped for the syntheses. | - Methods: Study selection - Table S10 |
| Information sources | 6 | Specify all databases, registers, websites, organisations, reference lists and other sources searched or consulted to identify studies. Specify the date when each source was last searched or consulted. | Methods: Search strategy |
| Search strategy | 7 | Present the full search strategies for all databases, registers and websites, including any filters and limits used. | Tables S2-S9 |
| Selection process | 8 | Specify the methods used to decide whether a study met the inclusion criteria of the review, including how many reviewers screened each record and each report retrieved, whether they worked independently, and if applicable, details of automation tools used in the process. | - Methods: Study selection - Table S10 |
| Data collection process | 9 | Specify the methods used to collect data from reports, including how many reviewers collected data from each report, whether they worked independently, any processes for obtaining or confirming data from study investigators, and if applicable, details of automation tools used in the process. | Methods: Data extraction |
| Data items | 10a | List and define all outcomes for which data were sought. Specify whether all results that were compatible with each outcome domain in each study were sought (e.g. for all measures, time points, analyses), and if not, the methods used to decide which results to collect. | Methods: Data extraction |
|  | 10b | List and define all other variables for which data were sought (e.g. participant and intervention characteristics, funding sources). Describe any assumptions made about any missing or unclear information. | Methods: Data extraction |
| Study risk of bias assessment | 11 | Specify the methods used to assess risk of bias in the included studies, including details of the tool(s) used, how many reviewers assessed each study and whether they worked independently, and if applicable, details of automation tools used in the process. | Methods: Quality assessment |
| Effect measures | 12 | Specify for each outcome the effect measure(s) (e.g. risk ratio, mean difference) used in the synthesis or presentation of results. | Methods: Data synthesis |
| Synthesis methods | 13a | Describe the processes used to decide which studies were eligible for each synthesis (e.g. tabulating the study intervention characteristics and comparing against the planned groups for each synthesis (item #5)). | Methods: Data synthesis |
|  | 13b | Describe any methods required to prepare the data for presentation or synthesis, such as handling of missing summary statistics, or data conversions. | Methods: Data synthesis |
|  | 13c | Describe any methods used to tabulate or visually display results of individual studies and syntheses. | Methods: Data synthesis |
|  | 13d | Describe any methods used to synthesize results and provide a rationale for the choice(s). If meta-analysis was performed, describe the model(s), method(s) to identify the presence and extent of statistical heterogeneity, and software package(s) used. | Methods: Data synthesis |
|  | 13e | Describe any methods used to explore possible causes of heterogeneity among study results (e.g. subgroup analysis, meta-regression). | Methods: Data synthesis |
|  | 13f | Describe any sensitivity analyses conducted to assess robustness of the synthesized results. | Methods: Data synthesis |
| Reporting bias assessment | 14 | Describe any methods used to assess risk of bias due to missing results in a synthesis (arising from reporting biases). | Methods: Quality assessment |
| Certainty assessment | 15 | Describe any methods used to assess certainty (or confidence) in the body of evidence for an outcome. | Methods: Quality assessment |
| **RESULTS** | | |  |
| Study selection | 16a | Describe the results of the search and selection process, from the number of records identified in the search to the number of studies included in the review, ideally using a flow diagram. | - Results: Search results - Figure 1 |
|  | 16b | Cite studies that might appear to meet the inclusion criteria, but which were excluded, and explain why they were excluded. | - Results: Search results - Table S12 |
| Study characteristics | 17 | Cite each included study and present its characteristics. | - Results: Overall summary of included studies - Table 1 |
| Risk of bias in studies | 18 | Present assessments of risk of bias for each included study. | Results: Synthesis of arguments |
| Results of individual studies | 19 | For all outcomes, present, for each study: (a) summary statistics for each group (where appropriate) and (b) an effect estimate and its precision (e.g. confidence/credible interval), ideally using structured tables or plots. | - Results: Overall summary of included studies - Table 1 |
| Results of syntheses | 20a | For each synthesis, briefly summarise the characteristics and risk of bias among contributing studies. | Results: Synthesis of arguments |
|  | 20b | Present results of all statistical syntheses conducted. If meta-analysis was done, present for each the summary estimate and its precision (e.g. confidence/credible interval) and measures of statistical heterogeneity. If comparing groups, describe the direction of the effect. | Results: Synthesis of arguments |
|  | 20c | Present results of all investigations of possible causes of heterogeneity among study results. | Results: Synthesis of arguments |
|  | 20d | Present results of all sensitivity analyses conducted to assess the robustness of the synthesized results. | Results: Synthesis of arguments |
| Reporting biases | 21 | Present assessments of risk of bias due to missing results (arising from reporting biases) for each synthesis assessed. | Results: Synthesis of arguments |
| Certainty of evidence | 22 | Present assessments of certainty (or confidence) in the body of evidence for each outcome assessed. | Results: Synthesis of arguments |
| **DISCUSSION** | | |  |
| Discussion | 23a | Provide a general interpretation of the results in the context of other evidence. | Discussion: Place in the literature |
|  | 23b | Discuss any limitations of the evidence included in the review. | Discussion: Strength and limitations |
|  | 23c | Discuss any limitations of the review processes used. | Discussion: Strength and limitations |
|  | 23d | Discuss implications of the results for practice, policy, and future research. | - Discussion: Suggestions for measuring effects of interventions for people with intellectual disabilities in economic evaluations - Discussion: Further research - Conclusions |
| **OTHER INFORMATION** | | |  |
| Registration and protocol | 24a | Provide registration information for the review, including register name and registration number, or state that the review was not registered. | Methods |
|  | 24b | Indicate where the review protocol can be accessed, or state that a protocol was not prepared. | Methods |
|  | 24c | Describe and explain any amendments to information provided at registration or in the protocol. | Methods |
| Support | 25 | Describe sources of financial or non-financial support for the review, and the role of the funders or sponsors in the review. | Funding statement |
| Competing interests | 26 | Declare any competing interests of review authors. | Conflict of interest disclosure |
| Availability of data, code and other materials | 27 | Report which of the following are publicly available and where they can be found: template data collection forms; data extracted from included studies; data used for all analyses; analytic code; any other materials used in the review. | Data availability statement |

^†^ *Template from:* Page MJ, McKenzie JE, Bossuyt PM, Boutron I, Hoffmann TC, Mulrow CD, et al. The PRISMA 2020 statement: an updated guideline for reporting systematic reviews. Bmj. 2021;372:n71.

## Table S2 Search strategy used in Medline (Ovid)

| **Search number** | **Query** |
| --- | --- |
| 1 | exp Child Development Disorders, Pervasive/ |
| 2 | exp Developmental Disabilities/ |
| 3 | exp Intellectual Disability/ |
| 4 | Learning Disabilities/ |
| 5 | (autis* or asperger* or aspie* or kanner* or rett*).tw. |
| 6 | ((learning or intellectual* or developmental) adj1 (disorder* or disable* or disabilit* or impair* or deficien* or difficult* or handicap* or subnormal* or "sub-normal*")).tw. |
| 7 | (Mental* adj (retard* or handicap* or disable* or disabilit* or impair* or deficien*)).tw. |
| 8 | (multipl* adj1 (handicap* or disab*)).tw. |
| 9 | (Down* syndrome* or Prader willi or labhart willi or royer syndrome* or Williams syndrome* or Fragile x or fraxe or fraxa or martin bell or marker x or Cri-du-Chat Syndrome or De Lange Syndrome or Rubinstein-Taybi Syndrome or Trisomy 13 or WAGR Syndrome or Angelman Syndrome).tw. |
| 10 | ((low or borderline or subnormal* or "sub-normal*") adj1 (intelligence or IQ)).tw. |
| 11 | or/1-10 |
| 12 | Health Care Costs/ |
| 13 | ec.fs. |
| 14 | Quality-Adjusted Life Years/ |
| 15 | (economic* adj6 (aspect* or model* or framework* or frame work* or method* or quality or technique* or outcome* or tool* or concept* or assess*)).tw. |
| 16 | ((preference or cost effectiveness or cost benefit) adj3 measur*).tw. |
| 17 | ((quality adjusted life year* or qaly*) and (alternative* or method* or utilit* or critici* or limitation* or drawback* or draw back* or problem* or disadvantage* or flaw* or weak* or issue* or disbenefit* or pitfall* or appropriate* or inappropriate* or suitab* or unsuitab*)).tw. |
| 18 | (quality adjusted life year* or qaly*).ti. |
| 19 | (quality of life adj3 (measur* or evaluat* or assess*)).tw. |
| 20 | (value based health or (valu* adj2 health state*)).tw. |
| 21 | (valu* adj3 health adj3 (outcome* or measur*)).tw. |
| 22 | or/12-21 |
| 23 | 11 and 22 |
| 24 | limit 23 to english language |

## Table S3 Search strategy used in Embase (Ovid)

| **Search number** | **Query** |
| --- | --- |
| 1 | developmental disorder/ |
| 2 | intellectual impairment/ |
| 3 | learning disorder/ |
| 4 | exp autism/ |
| 5 | (autis* or asperger* or aspie* or kanner* or rett*).tw. |
| 6 | ((learning or intellectual* or developmental) adj1 (disorder* or disable* or disabilit* or impair* or deficien* or difficult* or handicap* or subnormal* or "sub-normal*")).tw. |
| 7 | (Mental* adj (retard* or handicap* or disable* or disabilit* or impair* or deficien*)).tw. |
| 8 | (multipl* adj1 (handicap* or disab*)).tw. |
| 9 | (Down* syndrome* or Prader willi or labhart willi or royer syndrome* or Williams syndrome* or Fragile x or fraxe or fraxa or martin bell or marker x or Cri-du-Chat Syndrome or De Lange Syndrome or Rubinstein-Taybi Syndrome or Trisomy 13 or WAGR Syndrome or Angelman Syndrome).tw. |
| 10 | ((low or borderline or subnormal* or "sub-normal*") adj1 (intelligence or IQ)).tw. |
| 11 | or/1-10 |
| 12 | "health care cost"/ |
| 13 | quality adjusted life year/ |
| 14 | exp economics/ |
| 15 | (economic* adj6 (aspect* or model* or framework* or frame work* or method* or quality or technique* or outcome* or tool* or concept* or assess*)).tw. |
| 16 | ((preference or cost effectiveness or cost benefit) adj3 measur*).tw. |
| 17 | ((quality adjusted life year* or qaly*) and (alternative* or method* or utilit* or critici* or limitation* or drawback* or draw back* or problem* or disadvantage* or flaw* or weak* or issue* or disbenefit* or pitfall* or appropriate* or inappropriate* or suitab* or unsuitab*)).tw. |
| 18 | (quality adjusted life year* or qaly*).ti. |
| 19 | (quality of life adj3 (measur* or evaluat* or assess*)).tw. |
| 20 | (value based health or (valu* adj2 health state*)).tw. |
| 21 | (valu* adj3 health adj3 (outcome* or measur*)).tw. |
| 22 | or/12-21 |
| 23 | 11 and 22 |
| 24 | limit 23 to english language |

## Table S4 Search strategy used in CINAHL (EBSCO)

| **Search number** | **Query** |
| --- | --- |
| S1 | (MH "Child Development Disorders, Pervasive+") |
| S2 | (MH "Developmental Disabilities") |
| S3 | (MH "Intellectual Disability+") |
| S4 | (MH "Learning Disorders") |
| S5 | autis* or asperger* or aspie* or kanner* or rett* |
| S6 | (learning or intellectual* or developmental) N1 (disorder* or disable* or disabilit* or impair* or deficien* or difficult* or handicap* or subnormal* or "sub-normal*") |
| S7 | Mental* N1 (retard* or handicap* or disable* or disabilit* or impair* or deficien*) |
| S8 | multipl* N1 (handicap* or disab*) |
| S9 | "Down* syndrome*" or "Prader willi" or "labhart willi" or "royer syndrome*" or "Williams syndrome*" or "Fragile x" or fraxe or fraxa or "martin bell" or "marker x" or "Cri-du-Chat Syndrome" or "De Lange Syndrome" or "Rubinstein-Taybi Syndrome" or "Trisomy 13" or "WAGR Syndrome" or "Angelman Syndrome" |
| S10 | (low or borderline or subnormal* or "sub-normal*") N1 (intelligence or IQ) |
| S11 | S1 OR S2 OR S3 OR S4 OR S5 OR S6 OR S7 OR S8 OR S9 OR S10 |
| S12 | (MH "Health Care Costs") |
| S13 | MW "EC" |
| S14 | (MH "Quality-Adjusted Life Years") |
| S15 | economic* N6 (aspect* or model* or framework* or "frame work*" or method* or quality or technique* or outcome* or tool* or concept* or assess*) |
| S16 | (preference or "cost effectiveness" or "cost benefit") N3 measur* |
| S17 | ("quality adjusted life year*" or qaly*) and (alternative* or method* or utilit* or critici* or limitation* or drawback* or "draw back*" or problem* or disadvantage* or flaw* or weak* or issue* or disbenefit* or pitfall* or appropriate* or inappropriate* or suitab* or unsuitab*) |
| S18 | TI("quality adjusted life year*" or qaly*) |
| S19 | "quality of life" N3 (measur* or evaluat* or assess*) |
| S20 | "value based health" or (valu* N2 "health state*") |
| S21 | valu* N3 health N3 (outcome* or measur*) |
| S22 | "S12 OR S13 OR S14 OR S15 OR S16 OR S17 OR S18 OR S19 OR S20 OR S21 |
| S23 | S11 AND S22 Limiters - English Language |

## Table S5 Search strategy used in PsycINFO

| **Search number** | **Query** |
| --- | --- |
| S1 | DE "Developmental Disabilities" OR DE "Specific Language Impairment" |
| S2 | DE "Intellectual Development Disorder" OR DE "Anencephaly" OR DE "Crying Cat Syndrome" OR DE "Down's Syndrome" OR DE "Tay Sachs Disease" |
| S3 | DE "Learning Disorders" OR DE "Learning Disabilities" |
| S4 | DE "Autism Spectrum Disorders" OR DE "Autistic Traits" |
| S5 | autis* or asperger* or aspie* or kanner* or rett* |
| S6 | (learning or intellectual* or developmental) N1 (disorder* or disable* or disabilit* or impair* or deficien* or difficult* or handicap* or subnormal* or "sub-normal*") |
| S7 | Mental* N1 (retard* or handicap* or disable* or disabilit* or impair* or deficien*) |
| S8 | multipl* N1 (handicap* or disab*) |
| S9 | "Down* syndrome*" or "Prader willi" or "labhart willi" or "royer syndrome*" or "Williams syndrome*" or "Fragile x" or fraxe or fraxa or "martin bell" or "marker x" or "Cri-du-Chat Syndrome" or "De Lange Syndrome" or "Rubinstein-Taybi Syndrome" or "Trisomy 13" or "WAGR Syndrome" or "Angelman Syndrome" |
| S10 | (low or borderline or subnormal* or "sub-normal*") N1 (intelligence or IQ) |
| S11 | S1 OR S2 OR S3 OR S4 OR S5 OR S6 OR S7 OR S8 OR S9 OR S10 |
| S12 | DE "Health Care Costs" |
| S13 | DE "Economics" |
| S14 | economic* N6 (aspect* or model* or framework* or "frame work*" or method* or quality or technique* or outcome* or tool* or concept* or assess*) |
| S15 | (preference or "cost effectiveness" or "cost benefit") N3 measur* |
| S16 | ("quality adjusted life year*" or qaly*) and (alternative* or method* or utilit* or critici* or limitation* or drawback* or "draw back*" or problem* or disadvantage* or flaw* or weak* or issue* or disbenefit* or pitfall* or appropriate* or inappropriate* or suitab* or unsuitab*) |
| S17 | TI("quality adjusted life year*" or qaly*) |
| S18 | "quality of life" N3 (measur* or evaluat* or assess*) |
| S19 | "value based health" or (valu* N2 "health state*") |
| S20 | valu* N3 health N3 (outcome* or measur*) |
| S21 | S12 OR S13 OR S14 OR S15 OR S16 OR S17 OR S18 OR S19 OR S20 |
| S22 | S11 AND S21 |
| S23 | Limiters - English |

## Table S6 Search strategy used in the Cochrane Database of Systematic Reviews and Cochrane Central Register of Controlled Trials (Cochrane Library)

| **Search number** | **Query** |
| --- | --- |
| 1 | MeSH descriptor: [Child Development Disorders, Pervasive] explode all trees |
| 2 | MeSH descriptor: [Developmental Disabilities] explode all trees |
| 3 | MeSH descriptor: [Intellectual Disability] explode all trees |
| 4 | MeSH descriptor: [Learning Disabilities] explode all trees |
| 5 | (autis* or asperger* or aspie* or kanner* or rett*):ti,ab,kw |
| 6 | ((learning or intellectual* or developmental) NEAR/1 (disorder* or disable* or disabilit* or impair* or deficien* or difficult* or handicap* or subnormal* or sub-normal*)):ti,ab,kw |
| 7 | (Mental* NEAR/1 (retard* or handicap* or disable* or disabilit* or impair* or deficien*)):ti,ab,kw |
| 8 | (multipl* NEAR/1 (handicap* or disab*)):ti,ab,kw |
| 9 | ((Down* NEXT syndrome*) or "Prader willi" or "labhart willi" or "royer syndrome" or "Williams syndrome" or "Fragile x" or fraxe or fraxa or "martin bell" or "marker x" or "Cri-du-Chat Syndrome" or "De Lange Syndrome" or "Rubinstein-Taybi Syndrome" or "Trisomy 13" or "WAGR Syndrome" or "Angelman Syndrome"):ti,ab,kw |
| 10 | ((low or borderline or subnormal* or sub-normal*) NEAR/1 (intelligence or IQ)):ti,ab,kw |
| 11 | (Staniszewska et al., -#10) |
| 12 | MeSH descriptor: [Health Care Costs] explode all trees |
| 13 | MeSH descriptor: [] explode all trees and with qualifier(s): [economics - EC] |
| 14 | MeSH descriptor: [Quality-Adjusted Life Years] explode all trees |
| 15 | (economic* NEAR/6 (aspect* or model* or framework* or frame work* or method* or quality or technique* or outcome* or tool* or concept* or assess*)):ti,ab,kw |
| 16 | ((preference or "cost effectiveness" or "cost benefit") NEAR/3 measur*):ti,ab,kw |
| 17 | (("quality adjusted life year" or "quality adjusted life years" or qaly*) and (alternative* or method* or utilit* or critici* or limitation* or drawback* or "draw back" or "draw backs" or problem* or disadvantage* or flaw* or weak* or issue* or disbenefit* or pitfall* or appropriate* or inappropriate* or suitab* or unsuitab*)):ti,ab,kw |
| 18 | ("quality adjusted life year" or "quality adjusted life years" or qaly*):ti |
| 19 | ("quality of life" NEAR/3 (measur* or evaluat* or assess*)):ti,ab,kw |
| 20 | ("value based health"):ti,ab,kw |
| 21 | (valu* NEAR/2 ("health state" or "health states")):ti,ab,kw |
| 22 | (valu* NEAR/3 health NEAR/3 (outcome* or measur*)):ti,ab,kw |
| 23 | {OR #12-#22} |
| 24 | #11 AND #23 |

## Table S7 Search strategy used in the International Health Technology Assessment Database

| **Query** |
| --- |
| ((((valu* AND health AND (outcome* or measur*))[abs]) OR ("value based health") OR ("quality of life" AND (measur* or evaluat* or assess*)) OR ("quality adjusted life year*" or qaly*) OR (((preference or "cost effectiveness" or "cost benefit") AND measur*)[abs]) OR (economic* AND (aspect* or model* or framework* or frame work* or method* or quality or technique* or outcome* or tool* or concept* or assess*)) OR ("Quality-Adjusted Life Years"[mh]) OR ("Health Care Costs"[mh]))) AND (("Learning Disabilities"[mh]) OR ("Intellectual Disability"[mhe]) OR ("Developmental Disabilities"[mhe]) OR ("Child Development Disorders, Pervasive"[mhe])) - limit to English |
| (autis* or asperger* or aspie* or kanner* or rett*) AND ((((valu* AND health AND (outcome* or measur*))[abs]) OR ("value based health") OR ("quality of life" AND (measur* or evaluat* or assess*)) OR ("quality adjusted life year*" or qaly*) OR (((preference or "cost effectiveness" or "cost benefit") AND measur*)[abs]) OR (economic* AND (aspect* or model* or framework* or frame work* or method* or quality or technique* or outcome* or tool* or concept* or assess*)) OR ("Quality-Adjusted Life Years"[mh]) OR ("Health Care Costs"[mh]))) limit to English |
| ((learning or intellectual* or developmental) AND (disorder* or disable* or disabilit* or impair* or deficien* or difficult* or handicap* or subnormal* or "sub-normal*")) AND ((((valu* AND health AND (outcome* or measur*))[abs]) OR ("value based health") OR ("quality of life" AND (measur* or evaluat* or assess*)) OR ("quality adjusted life year*" or qaly*) OR (((preference or "cost effectiveness" or "cost benefit") AND measur*)[abs]) OR (economic* AND (aspect* or model* or framework* or frame work* or method* or quality or technique* or outcome* or tool* or concept* or assess*)) OR ("Quality-Adjusted Life Years"[mh]) OR ("Health Care Costs"[mh]))) limit to English |
| ("mentally retarded" or "mental retardation" or "mental handicap" or "mentally handicapped" or "mentally disabled" or "mental disability" or "mental disabilities" or "mental impairment" or "mentally impaired" or "mental deficiency" or "mentally deficient") AND ((((valu* AND health AND (outcome* or measur*))[abs]) OR ("value based health") OR ("quality of life" AND (measur* or evaluat* or assess*)) OR ("quality adjusted life year*" or qaly*) OR (((preference or "cost effectiveness" or "cost benefit") AND measur*)[abs]) OR (economic* AND (aspect* or model* or framework* or frame work* or method* or quality or technique* or outcome* or tool* or concept* or assess*)) OR ("Quality-Adjusted Life Years"[mh]) OR ("Health Care Costs"[mh]))) limit to English |
| (multiple AND (handicap* or disab*)) AND ((((valu* AND health AND (outcome* or measur*))[abs]) OR ("value based health") OR ("quality of life" AND (measur* or evaluat* or assess*)) OR ("quality adjusted life year*" or qaly*) OR (((preference or "cost effectiveness" or "cost benefit") AND measur*)[abs]) OR (economic* AND (aspect* or model* or framework* or frame work* or method* or quality or technique* or outcome* or tool* or concept* or assess*)) OR ("Quality-Adjusted Life Years"[mh]) OR ("Health Care Costs"[mh]))) limit to English |
| ("Down syndrome" or "downs syndrome" or "down's syndrome" or "Prader willi" or "labhart willi" or "royer syndrome" or "Williams syndrome" or "Fragile x" or fraxe or fraxa or "martin bell" or "marker x" or "Cri-du-Chat Syndrome" or "De Lange Syndrome" or "Rubinstein-Taybi Syndrome" or "Trisomy 13" or "WAGR Syndrome" or "Angelman Syndrome") AND ((((valu* AND health AND (outcome* or measur*))[abs]) OR ("value based health") OR ("quality of life" AND (measur* or evaluat* or assess*)) OR ("quality adjusted life year*" or qaly*) OR (((preference or "cost effectiveness" or "cost benefit") AND measur*)[abs]) OR (economic* AND (aspect* or model* or framework* or frame work* or method* or quality or technique* or outcome* or tool* or concept* or assess*)) OR ("Quality-Adjusted Life Years"[mh]) OR ("Health Care Costs"[mh]))) limit to English |
| ((low or borderline or subnormal* or "sub-normal") AND (intelligence or IQ)) AND ((((valu* AND health AND (outcome* or measur*))[abs]) OR ("value based health") OR ("quality of life" AND (measur* or evaluat* or assess*)) OR ("quality adjusted life year*" or qaly*) OR (((preference or "cost effectiveness" or "cost benefit") AND measur*)[abs]) OR (economic* AND (aspect* or model* or framework* or frame work* or method* or quality or technique* or outcome* or tool* or concept* or assess*)) OR ("Quality-Adjusted Life Years"[mh]) OR ("Health Care Costs"[mh]))) limit to English |

## Table S8 Search strategy used in the NHS Economic Evaluation Database

| **Search number** | **Query** |
| --- | --- |
| 1 | MeSH DESCRIPTOR Child Development Disorders, Pervasive EXPLODE ALL TREES |
| 2 | MeSH DESCRIPTOR Developmental Disabilities EXPLODE ALL TREES |
| 3 | MeSH DESCRIPTOR Intellectual Disability EXPLODE ALL TREES |
| 4 | MeSH DESCRIPTOR Learning Disabilities EXPLODE ALL TREES |
| 5 | (autis* or asperger* or aspie* or kanner* or rett*) IN NHSEED |
| 6 | ((learning or intellectual* or developmental) NEAR1 (disorder* or disable* or disabilit* or impair* or deficien* or difficult* or handicap* or subnormal* or sub-normal*)) IN NHSEED |
| 7 | (Mental* NEAR1 (retard* or handicap* or disable* or disabilit* or impair* or deficien*)) IN NHSEED |
| 8 | (multipl* NEAR1 (handicap* or disab*)) IN NHSEED |
| 9 | ("Down syndrome" or "downs syndrome" or "down's syndrome" or "Prader willi" or "labhart willi" or "royer syndrome" or "Williams syndrome" or "Fragile x" or fraxe or fraxa or "martin bell" or "marker x" or "Cri-du-Chat Syndrome" or "De Lange Syndrome" or "Rubinstein-Taybi Syndrome" or "Trisomy 13" or "WAGR Syndrome" or "Angelman Syndrome") IN NHSEED |
| 10 | ((low or borderline or subnormal* or sub-normal*) NEAR1 (intelligence or IQ)) IN NHSEED |
| 11 | #1 OR #2 OR #3 OR #4 OR #5 OR #6 OR #7 OR #8 OR #9 OR #10 |
| 12 | MeSH DESCRIPTOR Health Care Costs EXPLODE ALL TREES |
| 13 | MeSH DESCRIPTOR Quality-Adjusted Life Years EXPLODE ALL TREES |
| 14 | (economic* NEAR6 (aspect* or model* or framework* or frame work* or method* or quality or technique* or outcome* or tool* or concept* or assess*)) IN NHSEED |
| 15 | ((preference or cost effectiveness or cost benefit) NEAR3 measur*) IN NHSEED |
| 16 | (("quality adjusted life year*" or qaly*) AND (alternative* or method* or utilit* or critici* or limitation* or drawback* or "draw back*" or problem* or disadvantage* or flaw* or weak* or issue* or disbenefit* or pitfall* or appropriate* or inappropriate* or suitab* or unsuitab*)) IN NHSEED |
| 17 | ("quality of life" NEAR3 (measur* or evaluat* or assess*)) IN NHSEED |
| 18 | ("value based health") IN NHSEED |
| 19 | (valu* NEAR2 health state*) IN NHSEED |
| 20 | (valu* NEAR3 health NEAR3 (outcome* or measur*)) IN NHSEED |
| 21 | #12 OR #13 OR #14 OR #15 OR #16 OR #17 OR #18 OR #19 OR #20 |
| 22 | #11 AND #21 |

## Table S9 Websites and search string used in the grey literature searches

| **Websites** | **Search string** |
| --- | --- |
| ispor.org | (autism OR autistic OR asperger's OR "learning disabilities" OR "learning disorders" OR "intellectual disabilities" OR "intellectual disorders" OR "developmental disabilities" OR "developmental disorders" OR "mental disabilities") |
| healtheconomics.org |  |
| ohe.org |  |

## Table S10 Screening tool

| **Outcomes measures for economic evaluations and cost-effectiveness analyses of interventions for people with intellectual disabilities: a methodological systematic review** | |
| --- | --- |
| **Research aim:**  To systematically identify and assess studies which discuss the challenges, advantages and disadvantages associated to the use of outcome measures such as quality-adjusted life-year (QALY) and its alternatives in economic evaluations of interventions for people with intellectual disabilities. | |
| **Key elements** | |
| **Population** | **Included:**   - General population with intellectual disabilities. |
|  | **Excluded:**   - General population not affected by intellectual disabilities. |
| **Interventions** | **Included**:   - Any intervention delivered for people with intellectual disabilities. |
|  | **Excluded**:   - Any intervention which is not targeted to people with intellectual disabilities. |
| **Settings** | **Any** |
| **Outcomes** | **Included:**   - Any study including a discussion of the theoretical and empirical challenges, advantages and disadvantages associated with the measurement, valuation and use of outcome measures (e.g. QALY and its alternatives) for economic evaluations of interventions for people with intellectual disabilities. |
|  | **Excluded:**   - Any study that does not include a discussion of the methodological challenges attached to the use of outcome measure for economic evaluations of interventions for people with intellectual disabilities (e.g. studies which only list or summarise the outcome measures used in economic evaluations). |
| **Comparison** | **Any** |
| **Study design** | **Included:**   - Empirical studies:   - systematic reviews (and meta-analyses) of economic evaluations;   - scoping reviews of economic evaluations;   - qualitative reviews of economic evaluations;   - narrative reviews of economic evaluations;   - full applied economic evaluations (i.e. which consider both the costs and the consequences of interventions for people with intellectual disabilities and their comparators);   - observational studies (e.g. cohort and case-control studies);   - interventional studies (e.g. pre-post study, non-randomised and randomised controlled trials); - Non-empirical studies: - theoretical or conceptual papers; - economic guidelines and checklists; - position papers; - editorials; - commentaries; - letters. |
|  | **Excluded**:   - Abstracts. |
| **Limits** | English language studies only. |

## Table S11 Guidance for Reporting Involvement of Patients and Public (GRIPP2) table^†^

| **Section and topic** | **Item** |
| --- | --- |
| 1: Aim | To identify, assess and synthesise the arguments in the literature on how the effects of interventions for people with intellectual disabilities could be measured in economic evaluations. |
| 2: Methods | We recruited one member of the public (NT) after circulating an expression of interest call within the Public Advisers’ Forum of the National Institute for Health Research Applied Research Collaboration North West Coast (NIHR ARC NWC). The call specified the aim, outline and timescales of the planned systematic review, together with the expected input from the member of the public. In particular, we sought input in terms of reading, reviewing and commenting on the draft protocol and report from the systematic review to inform the presentation, interpretation and application of the results.  The call also detailed the type of payment for the member of the public’s input. |
| 3: Study results | In an initial meeting between one of the co-authors (VB) and NT  the systematic review was discussed, and reciprocal expectations set out in more detail. The tasks and related time commitment for NT, as well as the support available from VB and the other co-authors, were outlined and agreed.  NT was then asked to provide her input on two key stages of the systematic review process: (i) development of the protocol of the systematic review; (ii) and draft of the manuscript once the systematic review was completed.   1. NT feedback on the draft protocol of the systematic review mainly covered the following:  - The clarity of the systematic review process and focus; - The description of the target population under examination (i.e. people with intellectual disabilities); - The inclusion of easy read outputs for service users in the dissemination strategy; - General wording.   Her valuable suggestions were incorporated in the final version of the protocol which was then submitted and registered on  PROSPERO, with NT included as one of the co-authors.  During the systematic review process, one of the co-authors (VB) sent regular updates to NT to provide information about the progress of the work and any adjustments in the expected timescales.   1. The feedback by NT on the draft of the manuscript focused on:  - Checking the description of ‘intellectual disability’ and the target population, providing suggestions for improvement; - Improving general wording and terminology related to intellectual disabilities; - Reviewing this table outlining her involvement and the Patient and Public Involvement process.   The corrections and suggestions provided by NT were incorporated in the final draft of the manuscript, which was improved thanks to her feedback. |
| 4: Discussion and conclusions | The input provided by NT helped the development of the systematic review and its finalisation along three main strands:  1. Valuable comments by NT improved the way the term ‘intellectual disability’ and the target population were described, in order to seek consistency and clarity across the protocol and manuscript;  2. Corrections on general wording and terminology helped understand which parts required further work to achieve appropriate clarity in the work and findings described;  3. NT also suggested ways to improve the dissemination strategy through the inclusion of easy read formats, which we are looking to produce to widen the diffusion of the findings. |
| 5: Reflections/critical perspective | This systematic review was enriched by the involvement and input of NT, which were unique in our team. Her contribution to improving the clarity of the process and messages conveyed, as well as her suggestions on the dissemination strategy, represent key support to this work, which was undoubtedly worth seeking.  Besides the outputs of the Patient and Public Involvement, its process was characterised by regular communication with NT. This is crucial in order to ensure that NT was continually informed about the progress of the systematic review, and about any changes to the expected timescale. This line of communication will be kept open during the publication and dissemination processes, in order to ensure that the input by NT will inform future activities. |

^†^ *Template from:* Staniszewska S, Brett J, Simera I, Seers K, Mockford C, Goodlad S, et al. GRIPP2 reporting checklists: tools to improve reporting of patient and public involvement in research. Res Involv Engagem. 2017;3:13.

## Table S12 Records excluded following full-text screening (n=49)

| **First author (year)** | **Reason for exclusion** |  |
| --- | --- | --- |
|  | **No detail on measurement, valuation, choice and use of outcome measures for economic evaluations** | **Little or marginal detail on measurement, valuation, choice and use of outcome measures for economic evaluations** |
| Angjellari-Dajci (2013) (Angjellari-Dajci, Lawless, Stachura, Wood, & DiBattisto, 2013) |  | 🗸 |
| Arkkila (2008) (Arkkila, Rasanen, Roine, Sintonen, & Vilkman, 2008) |  | 🗸 |
| Arkkila (2011) (Arkkila et al., 2011) |  | 🗸 |
| Bouwmans (2014) (Bouwmans et al., 2014) |  | 🗸 |
| Brazier (2008) (Brazier, 2008) |  | 🗸 |
| Burström (2014) (Burstrom, Bartonek, Brostrom, Sun, & Egmar, 2014) |  | 🗸 |
| Cavazza (2016) (Cavazza et al., 2016) | 🗸 |  |
| Chevreul (2015) (Chevreul, Berg Brigham, Brunn, des Portes, & Network, 2015) |  | 🗸 |
| Chevreul (2016) (Chevreul et al., 2016) |  | 🗸 |
| Chisholm (1997) (Chisholm, Healey, & Knapp, 1997) |  | 🗸 |
| Christensen (2017) (Christensen, MacIntosh, Switzer, & Fehlings, 2017) | 🗸 |  |
| Crisp (1991) (Crisp, 1991) |  | 🗸 |
| de Sonneville (2014) (de Sonneville-Koedoot, Stolk, Raat, Bouwmans-Frijters, & Franken, 2014) |  | 🗸 |
| de Sonneville (2015) (de Sonneville-Koedoot, Bouwmans, Franken, & Stolk, 2015) |  | 🗸 |
| Domellof (2014) (Domellof, Hedlund, & Odman, 2014) |  | 🗸 |
| Donaldson (1988) (Donaldson, Atkinson, Bond, & Wright, 1988) |  | 🗸 |
| Griffin (2008) (Griffin, Weatherly, Richardson, & Drummond, 2008) |  | 🗸 |
| Guyatt (1993) (Guyatt, Feeny, & Patrick, 1993) | 🗸 |  |
| Hammerman (1983) (Hammerman & Maikowski, 1983) |  |  |
| Hernandez-Villafuerte (2018) (Hernandez-Villafuerte et al., 2019) |  | 🗸 |
| Hoving (2008) (Hoving et al., 2008) | 🗸 |  |
| Knapp (2007) (M. Knapp & Mangalore, 2007) | 🗸 |  |
| Knapp (2014) (Martin Knapp & Buescher, 2014) |  | 🗸 |
| Knobbe (1995) (Knobbe, Carey, Rhodes, & Horner, 1995) |  | 🗸 |
| Jog (2016) (Jog et al., 2016) |  | 🗸 |
| Lamsal (2018) (Lamsal, Dutton, & Zwicker, 2018) | 🗸 |  |
| Landfelt (2016) (Landfeldt et al., 2016) | 🗸 |  |
| Maia (2016) (Maia et al., 2016) | 🗸 |  |
| Matza (2005) (Matza, Secnik, Mannix, & Sallee, 2005) |  | 🗸 |
| Mok (2014) (Mok et al., 2014) | 🗸 |  |
| Payakachat (2014) [5] |  | 🗸 |
| Peasgood (2016) (Peasgood et al., 2016) |  | 🗸 |
| Perez Sousa (2017) (Perez Sousa, Olivares Sanchez-Toledo, & Gusi Fuerte, 2017) | 🗸 |  |
| Petrou (2009) (Petrou & Kupek, 2009) | 🗸 |  |
| Petrou (2010) (Petrou et al., 2010) |  | 🗸 |
| Petrou (2013) (Petrou, Johnson, Wolke, & Marlow, 2013) |  | 🗸 |
| Rosenbaum (2007) (Rosenbaum, Livingston, Palisano, Galuppi, & Russell, 2007) |  | 🗸 |
| Secnik (2005) (Secnik et al., 2005) |  | 🗸 |
| Sipila (2010) (Sipila et al., 2010) | 🗸 |  |
| Stade (2006) (Stade, Stevens, Ungar, Beyene, & Koren, 2006) |  | 🗸 |
| Stant (2007) (Stant, Buskens, Jenner, Wiersma, & TenVergert, 2007) |  | 🗸 |
| Tilford (2012) (Tilford et al., 2012) |  | 🗸 |
| Tilford (2015) (Tilford et al., 2015) |  | 🗸 |
| van der Kolk (2014) (van der Kolk et al., 2014) |  | 🗸 |
| van Ijzendoorn (2020) (van Ijzendoorn & Bakermans-Kranenburg, 2020) |  | 🗸 |
| van Steensel (2012) (F. J. van Steensel, Bogels, & Dirksen, 2012) | 🗸 |  |
| van Steensel (2014) (F. J. A. Van Steensel, Dirksen, & Bogels, 2014) | 🗸 |  |
| Vermeulen (2017) (Vermeulen, Jansen, Buskens, Knorth, & Reijneveld, 2017) |  | 🗸 |
| Willems (2009) (Willems et al., 2009) |  | 🗸 |
| Young (2010) (Young et al., 2010) |  | 🗸 |
| **n(%)** | 14(28.57) | 35(71.43) |

# References

Angjellari-Dajci, F., Lawless, W. F., Stachura, M. E., Wood, E. A., & DiBattisto, C. (2013). Economic evaluations for service delivery in autism spectrum disorders: Benefit-cost analysis for emerging telehealth systems. In *Handbook of Research on ICTs and Management Systems for Improving Efficiency in Healthcare and Social Care* (Vol. 1-2, pp. 16-42).

Arkkila, E., Räsänen, P., Roine, R. P., Sintonen, H., Saar, V., & Vilkman, E. (2011). Health-related quality of life of children with specific language impairment aged 8-11. *Folia Phoniatrica et Logopaedica, 63*(1), 27-35. doi:10.1159/000319735

Arkkila, E., Rasanen, P., Roine, R. P., Sintonen, H., & Vilkman, E. (2008). Health-related quality of life of adults with childhood diagnosis of specific language impairment. *Folia Phoniatrica et Logopedica, 60*(5), 233-240. doi:10.1159/000151325

Bouwmans, C., van der Kolk, A., Oppe, M., Schawo, S., Stolk, E., van Agthoven, M., . . . van Roijen, L. (2014). Validity and responsiveness of the EQ-5D and the KIDSCREEN-10 in children with ADHD. *European Journal of Health Economics, 15*(9), 967-977. doi:10.1007/s10198-013-0540-x

Brazier, J. (2008). Measuring and valuing mental health for use in economic evaluation. *Journal of Health Services Research & Policy, 13*, 70-75. doi:10.1258/jhsrp.2008.008015

Burstrom, K., Bartonek, A., Brostrom, E. W., Sun, S., & Egmar, A. C. (2014). EQ-5D-Y as a health-related quality of life measure in children and adolescents with functional disability in Sweden: testing feasibility and validity. *Acta Paediatrica, 103*(4), 426-435. doi:10.1111/apa.12557

Cavazza, M., Kodra, Y., Armeni, P., De Santis, M., Lopez-Bastida, J., Linertova, R., . . . Network, B.-R. R. (2016). Social/economic costs and health-related quality of life in patients with Duchenne muscular dystrophy in Europe. *Eur J Health Econ, 17 Suppl 1*, 19-29. doi:10.1007/s10198-016-0782-5

Chevreul, K., Berg Brigham, K., Brunn, M., des Portes, V., & Network, B.-R. R. (2015). Fragile X syndrome: economic burden and health-related quality of life of patients and caregivers in France. *Journal of Intellectual Disability Research, 59*(12), 1108-1120. doi:<https://dx.doi.org/10.1111/jir.12215>

Chevreul, K., Berg Brigham, K., Clement, M. C., Poitou, C., Tauber, M., & Members of the, B.-R. D. R. N. l. i. t. O. A. (2016). Economic burden and health-related quality of life associated with Prader-Willi syndrome in France. *Journal of Intellectual Disability Research, 60*(9), 879-890. doi:<https://dx.doi.org/10.1111/jir.12288>

Chisholm, D., Healey, A., & Knapp, M. (1997). QALYs and mental health care. *Social Psychiatry and Psychiatric Epidemiology, 32*(2), 68-75. doi:10.1007/bf00788923

Christensen, R., MacIntosh, A., Switzer, L., & Fehlings, D. (2017). Change in pain status in children with cerebral palsy. *Developmental Medicine & Child Neurology, 59*(4), 374-379. doi:10.1111/dmcn.13328

Crisp, R. (1991). QALYs and the mentally handicapped. *Bulletin of Medical Ethics. No, 67*, 13-16.

de Sonneville-Koedoot, C., Bouwmans, C., Franken, M. C., & Stolk, E. (2015). Economic evaluation of stuttering treatment in preschool children: The RESTART-study. *J Commun Disord, 58*, 106-118. doi:10.1016/j.jcomdis.2015.10.006

de Sonneville-Koedoot, C., Stolk, E. A., Raat, H., Bouwmans-Frijters, C., & Franken, M. C. (2014). Health-related quality of life of preschool children who stutter. *J Fluency Disord, 42*, 1-12. doi:10.1016/j.jfludis.2014.09.001

Domellof, E., Hedlund, L., & Odman, P. (2014). Health-related quality of life of children and adolescents with functional disabilities in a northern Swedish county. *Quality of Life Research, 23*(6), 1877-1882. doi:10.1007/s11136-013-0613-4

Donaldson, C., Atkinson, A., Bond, J., & Wright, K. (1988). Should QALYs be programme-specific? *Journal of Health Economics, 7*(3), 239-257.

Griffin, S. C., Weatherly, H. L. A., Richardson, G. A., & Drummond, M. F. (2008). Methodological issues in undertaking independent cost-effectiveness analysis for NICE: The case of therapies for ADHD. *The European Journal of Health Economics, 9*(2), 137-145. doi:10.1007/s10198-007-0052-7

Guyatt, G. H., Feeny, D. H., & Patrick, D. L. (1993). Measuring health-related quality of life. *Annals of Internal Medicine, 118*(8), 622-629. doi:10.7326/0003-4819-118-8-199304150-00009

Hammerman, S. R., & Maikowski, S. (1983). The economics of disability from an international perspective. *Annual Review of Rehabilitation, 3*, 178-202.

Hernandez-Villafuerte, K., Zamora, B., Feng, Y., Parkin, D., Devlin, N., & Towse, A. (2019). Exploring variations in the opportunity cost cost-effectiveness threshold by clinical area: Results from a feasibility study.

Hoving, M. A., Evers, S. M., Ament, A. J., van Raak, E. P., Vles, J. S., & Dutch Study Group on Child, S. (2008). Intrathecal baclofen therapy in children with intractable spastic cerebral palsy: a cost-effectiveness analysis. *Developmental Medicine & Child Neurology, 50*(6), 450-455. doi:10.1111/j.1469-8749.2008.02059.x

Jog, M., Wein, T., Bhogal, M., Dhani, S., Miller, R., Ismail, F., . . . Trentin, G. (2016). Real-World, Long-Term Quality of Life Following Therapeutic OnabotulinumtoxinA Treatment. *Can J Neurol Sci, 43*(5), 687-696. doi:10.1017/cjn.2016.262

Knapp, M., & Buescher, A. (2014). Economic aspects of autism. In F. R. Volkmar, S. J. Rogers, R. Paul, & K. A. Pelphrey (Eds.), *Handbook of autism and pervasive developmental disorders: Assessment, interventions, and policy., Volume 2, 4th ed.* (pp. 1089-1106). Hoboken, NJ: John Wiley & Sons, Inc.

Knapp, M., & Mangalore, R. (2007). "The trouble with QALYs ...". *Epidemiologia E Psichiatria Sociale-an International Journal for Epidemiology and Psychiatric Sciences, 16*(4), 289-293. doi:10.1017/s1121189x00002451

Knobbe, C. A., Carey, S. P., Rhodes, L., & Horner, R. H. (1995). Benefit-cost analysis of community residential versus institutional services for adults with severe mental retardation and challenging behaviors. *American Journal of Mental Retardation, 99*(5), 533-541.

Lamsal, R., Dutton, D. J., & Zwicker, J. D. (2018). Using the ages and stages questionnaire in the general population as a measure for identifying children not at risk of a neurodevelopmental disorder. *BMC Pediatrics, 18*, 9. doi:10.1186/s12887-018-1105-z

Landfeldt, E., Lindgren, P., Bell, C. F., Guglieri, M., Straub, V., Lochmuller, H., & Bushby, K. (2016). Health-related quality of life in patients with Duchenne muscular dystrophy: a multinational, cross-sectional study. *Developmental Medicine & Child Neurology, 58*(5), 508-515. doi:10.1111/dmcn.12938

Maia, C. R., Stella, S. F., Wagner, F., Pianca, T. G., Krieger, F. V., Cruz, L. N., . . . Polanczyk, C. A. (2016). Cost-utility analysis of methylphenidate treatment for children and adolescents with ADHD in Brazil. *Revista Brasileira de Psiquiatria, 38*(1), 30-38. doi:10.1590/1516-4446-2014-1516

Matza, L. S., Secnik, K., Mannix, S., & Sallee, F. R. (2005). Parent-proxy EQ-5D ratings of children with attention-deficit hyperactivity disorder in the US and the UK. *PharmacoEconomics, 23*(8), 777-790. doi:10.2165/00019053-200523080-00004

Mok, W. K. Y., Wong, W. H.-S., Mok, G. T. K., Chu, Y. W. Y., Ho, F. K. W., Chow, C. B., . . . Chung, B. H.-Y. (2014). Validation and application of health utilities index in Chinese subjects with down syndrome. *Health and Quality of Life Outcomes, 12*(1), 144. doi:10.1186/s12955-014-0144-x

Peasgood, T., Bhardwaj, A., Biggs, K., Brazier, J. E., Coghill, D., Cooper, C. L., . . . Sonuga-Barke, E. J. (2016). The impact of ADHD on the health and well-being of ADHD children and their siblings. *Eur Child Adolesc Psychiatry, 25*(11), 1217-1231. doi:10.1007/s00787-016-0841-6

Perez Sousa, M. A., Olivares Sanchez-Toledo, P. R., & Gusi Fuerte, N. (2017). Parent-child discrepancy in the assessment of health- related quality of life using the EQ-5D-Y questionnaire. *Arch Argent Pediatr, 115*(6), 541-546. doi:10.5546/aap.2017.eng.541

Petrou, S., Johnson, S., Wolke, D., Hollis, C., Kochhar, P., & Marlow, N. (2010). Economic costs and preference-based health-related quality of life outcomes associated with childhood psychiatric disorders. *British Journal of Psychiatry, 197*(5), 395-404. doi:<https://dx.doi.org/10.1192/bjp.bp.110.081307>

Petrou, S., Johnson, S., Wolke, D., & Marlow, N. (2013). The association between neurodevelopmental disability and economic outcomes during mid-childhood. *Child: Care, Health & Development, 39*(3), 345-357. doi:<https://dx.doi.org/10.1111/j.1365-2214.2012.01368.x>

Petrou, S., & Kupek, E. (2009). Estimating preference-based health utilities index mark 3 utility scores for childhood conditions in England and Scotland. *Medical Decision Making, 29*(3), 291-303. doi:<http://dx.doi.org/10.1177/0272989X08327398>

Rosenbaum, P. L., Livingston, M. H., Palisano, R. J., Galuppi, B. E., & Russell, D. J. (2007). Quality of life and health-related quality of life of adolescents with cerebral palsy. *Developmental Medicine & Child Neurology, 49*(7), 516-521.

Secnik, K., Matza, L. S., Cottrell, S., Edgell, E., Tilden, D., & Mannix, S. (2005). Health state utilities for childhood attention-deficit/hyperactivity disorder based on parent preferences in the United kingdom. *Medical Decision Making, 25*(1), 56-70. doi:10.1177/0272989X04273140

Sipila, I., Sintonen, H., Hietanen, H., Apajasalo, M., Alanne, S., Viita, A. M., & Leinonen, E. (2010). Long-term effects of growth hormone therapy on patients with Prader-Willi syndrome. *Acta Paediatrica, 99*(11), 1712-1718. doi:<https://dx.doi.org/10.1111/j.1651-2227.2010.01904.x>

Stade, B. C., Stevens, B., Ungar, W. J., Beyene, J., & Koren, G. (2006). Health-related quality of life of Canadian children and youth prenatally exposed to alcohol. *Health & Quality of Life Outcomes, 4*, 81. doi:10.1186/1477-7525-4-81

Staniszewska, S., Brett, J., Simera, I., Seers, K., Mockford, C., Goodlad, S., . . . Tysall, C. (2017). GRIPP2 reporting checklists: tools to improve reporting of patient and public involvement in research. *Res Involv Engagem, 3*, 13. doi:10.1186/s40900-017-0062-2

Stant, A. D., Buskens, E., Jenner, J. A., Wiersma, D., & TenVergert, E. M. (2007). Cost-effectiveness analysis in severe mental illness: Outcome measures selection. *Journal of Mental Health Policy and Economics, 10*(2), 101-108.

Tilford, J. M., Payakachat, N., Kovacs, E., Pyne, J. M., Brouwer, W., Nick, T. G., . . . Kuhlthau, K. A. (2012). Preference-based health-related quality-of-life outcomes in children with autism spectrum disorders: a comparison of generic instruments. *PharmacoEconomics, 30*(8), 661-679. doi:<https://dx.doi.org/10.2165/11597200-000000000-00000>

Tilford, J. M., Payakachat, N., Kuhlthau, K. A., Pyne, J. M., Kovacs, E., Bellando, J., . . . Frye, R. E. (2015). Treatment for Sleep Problems in Children with Autism and Caregiver Spillover Effects. *Journal of Autism & Developmental Disorders, 45*(11), 3613-3623. doi:<https://dx.doi.org/10.1007/s10803-015-2507-5>

van der Kolk, A., Bouwmans, C. A., Schawo, S. J., Buitelaar, J. K., van Agthoven, M., & Hakkaart-van Roijen, L. (2014). Association between quality of life and treatment response in children with attention Deficit Hyperactivity Disorder and their parents. *The Journal of Mental Health Policy & Economics, 17*(3), 119-129.

van Ijzendoorn, M. H., & Bakermans-Kranenburg, M. J. (2020). Problematic cost-utility analysis of interventions for behavior problems in children and adolescents. In B. Barbot (Ed.), *Transition & Development* (Vol. 172, pp. 89-102). San Francisco: Wiley Periodicals.

van Steensel, F. J., Bogels, S. M., & Dirksen, C. D. (2012). Anxiety and quality of life: clinically anxious children with and without autism spectrum disorders compared. *Journal of Clinical Child & Adolescent Psychology, 41*(6), 731-738. doi:<https://dx.doi.org/10.1080/15374416.2012.698725>

Van Steensel, F. J. A., Dirksen, C. D., & Bogels, S. M. (2014). Cost-effectiveness of cognitive-behavioral therapy versus treatment as usual for anxiety disorders in children with autism spectrum disorder. *Research in Autism Spectrum Disorders, 8*(2), 127-137. doi:<http://dx.doi.org/10.1016/j.rasd.2013.11.001>

Vermeulen, K. M., Jansen, D., Buskens, E., Knorth, E. J., & Reijneveld, S. A. (2017). Serious child and adolescent behaviour disorders; a valuation study by professionals, youth and parents. *BMC Psychiatry, 17*(1), 208. doi:10.1186/s12888-017-1363-6

Willems, D. C., Joore, M. A., Nieman, F. H., Severens, J. L., Wouters, E. F., & Hendriks, J. J. (2009). Using EQ-5D in children with asthma, rheumatic disorders, diabetes, and speech/language and/or hearing disorders. *International Journal of Technology Assessment in Health Care, 25*(3), 391-399. doi:10.1017/S0266462309990171

Young, N. L., Rochon, T. G., McCormick, A., Law, M., Wedge, J. H., & Fehlings, D. (2010). The health and quality of life outcomes among youth and young adults with cerebral palsy. *Archives of Physical Medicine & Rehabilitation, 91*(1), 143-148. doi:10.1016/j.apmr.2009.08.152
